# A Cluster of Three Cases of* Hantavirus* Pulmonary Syndrome among Canadian Military Personnel

**DOI:** 10.1155/2016/2757969

**Published:** 2016-04-10

**Authors:** Leighanne O. Parkes, Trong Tien Nguyen, Jean Longtin, Marie-Claude Beaudoin, Julie Bestman-Smith, Donald C. Vinh, Guy Boivin, Vivian G. Loo

**Affiliations:** ^1^Division of Infectious Diseases and Department of Medical Microbiology, McGill University Health Centre, McGill University, 1001 Boulevard Décarie, Montréal, QC, Canada H4A 3J1; ^2^Infectious Diseases Research Center of the CHU de Québec, Université Laval, 2705 Boulevard Laurier, Québec, QC, Canada G1V 4G2; ^3^Laboratoire de Santé Publique du Québec, 20045 Chemin Ste-Marie, Sainte-Anne-de-Bellevue, QC, Canada H9X 3R5; ^4^CHU de Québec-Université Laval, Hôpital de l'Enfant Jésus, 1401 18e rue, Québec, QC, Canada G1J 1Z4

## Abstract

*Hantavirus* pulmonary syndrome (HPS) is a rare illness in eastern Canada. We present three cases of HPS among military personnel in Quebec. The three cases shared a common exposure to mouse excreta while engaged in military training in Alberta, a western province of Canada.

## 1. Introduction

The* Hantavirus* genus (family Bunyaviridae) contains more than 20 species, each typically associated with a single rodent reservoir, resulting in geographically distinct human diseases. The clinical spectrum of* Hantavirus* infection includes nephropathia epidemica, hemorrhagic fever with renal syndrome, and* Hantavirus* pulmonary syndrome (HPS) [1]. HPS presents with a viral prodrome followed by fulminant noncardiogenic pulmonary edema.

**Figure 1:**
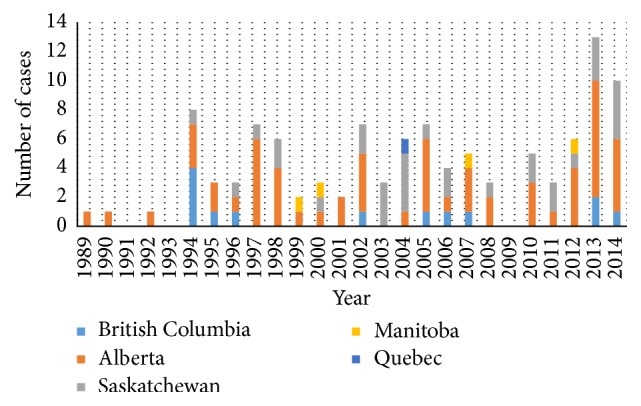
Number of* Hantavirus* cases in Canada from 1989 to 2014. Reproduced and adapted with permission from Drebot et al.

HPS was first described in 1993 in South-Western USA. Canada's first case was recognized in 1994. As of December 31, 2014, 109 Canadian cases of HPS have been laboratory-confirmed, averaging 5 cases yearly, mainly in the western provinces of Canada (Figure 1) [2]. The Sin Nombre strain is most commonly associated with HPS in Canada and the United States, reflecting the ubiquitous distribution of its reservoir, the deer mouse,* Peromyscus maniculatus*.

Until recently, only a single laboratory-confirmed case of* Hantavirus* infection has been reported in 2004 from eastern Canada, in the province of Quebec [3]. In this report, we describe an unusual cluster of three new cases of HPS diagnosed in Quebec in June and July of 2015.

## 2. Patient 1

In late June 2015, a healthy 22-year-old male presented to a community hospital in Montreal, Quebec, with vomiting, abdominal pain, and diarrhea for two days. He had returned from four weeks of military training in Alberta from April to May 2015. He was febrile at 39.3°C and tachycardic but his exam was otherwise unremarkable. Broad spectrum antibiotics were initiated for possible sepsis. Laboratory investigations are shown in Table 1.

**Figure 2:**
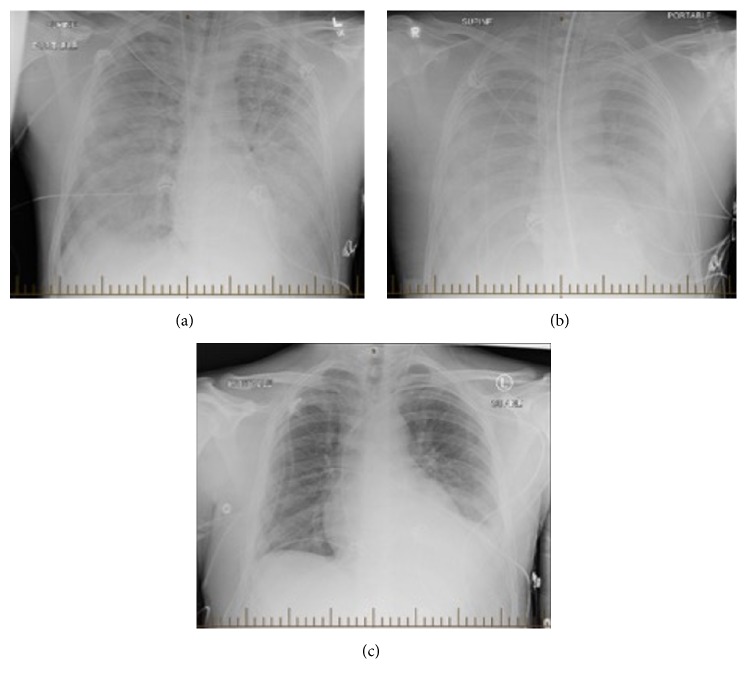
Radiographic progression of pulmonary disease in patient 1. (a) Chest radiograph on presentation; (b) chest radiograph on day two of admission following ECMO; (c) chest radiograph on day 10 of admission following extubation.

The following day, the patient rapidly deteriorated, requiring intubation for hypoxic respiratory failure. Chest radiograph revealed bilateral pulmonary infiltrates with effusions (Figure 2). Due to refractory hypoxia and hypoperfusion, he was transferred to a tertiary care center ICU for extracorporeal membrane oxygenation (ECMO). Blood, urine, and stool cultures were negative. Respiratory virus PCR from nasopharyngeal swab was also negative for the respiratory pathogens. The patient was extubated following gradual improvement and he left hospital without sequelae.

Based on his epidemiological risk factors,* Hantavirus* serology was performed and was positive for both* Hantavirus* Sin Nombre IgG and IgM. This prompted public health notification. PCR targeting the conserved region of* Hantavirus* M segment detected the presence of* Hantavirus* from a nasopharyngeal swab sample that was procured on day six of illness. Further* Hantavirus* typing was performed by direct sequencing of the amplicon with sequence analysis using NCBI Basic Local Alignment Search Tool (BLAST) demonstrating similarity to Sin Nombre reference strains from Montana and Alberta.

## 3. Patient 2

In late June 2015, a 32-year-old male known for asthma and smoking presented to a Quebec City hospital. He had participated in the same military training as patient 1, during the same time period. Two days prior to admission, he developed nonproductive cough, fever, shortness of breath, myalgias, and headache. The patient was initially afebrile but tachycardic and hypotensive (BP 85/47 mmHg). Chest radiograph revealed multifocal interstitial opacities. Levofloxacin was given for possible atypical pneumonia. Laboratory investigations are shown in Table 1.

The patient became more hypoxic over 48 hours and was intubated. His antimicrobial regimen was expanded. Bronchoalveolar lavage (BAL) was negative for bacterial and fungal cultures,* Pneumocystis*,* Mycoplasma*, and* Chlamydophila* PCR, as was urinary legionella antigen. The patient gradually improved and was extubated without further sequelae.

Prompted by the public health alert, serology was performed, confirming positive* Hantavirus* IgG and IgM.* Hantavirus* PCR performed on the BAL specimen was negative.

## 4. Patient 3

In early July 2015, a healthy 30-year-old male presented to another Quebec City hospital with malaise, fever, cough, and dyspnea for three days. He developed vomiting prior to presenting. He had participated in the same military training at the same time as the previous two patients. He was febrile at 40.8°C and hypoxic with an oxygen saturation of 93% on room air. Chest radiograph revealed bilateral interstitial opacities with effusions. He received broad spectrum antibiotics for presumed complicated pneumonia. Laboratory investigations are shown in Table 1. The patient required noninvasive positive pressure ventilation (NIPPV) for hypoxia.

Cultures from BAL revealed normal flora and were negative for* Pneumocystis*,* Legionella*, mycobacteria, or fungi. Blood cultures and* Histoplasma* urinary antigen were negative. This patient did not require intubation and rapidly improved over several days.

*Hantavirus* serology was IgG positive at 1/400, but IgM equivocal. Repeat serology revealed a rise in IgG titre to 1/1600.* Hantavirus* PCR performed on the BAL specimen was negative.

## 5. Epidemiology

The three patients were enrolled in the Canadian Armed Forces and based at Valcartier, Quebec. There was no history of foreign deployment. They had returned from a large-scale military training exercise in Alberta that ran from April 20 until May 23, 2015, and involved 6,750 military personnel from Canada, the United States, and the United Kingdom. Patients reported mouse sightings and rodent excreta in the campsite and near their tents. They were exposed to aerosolized soil through activities such as military vehicle driving, trench excavation, live fire field exercises, and detonation of ammunition shells. Although the patients were from different battalions, they participated in a common field exercise during their final week of training. The last case of* Hantavirus* from this region was reported in 1999 (M. Maher, pers. comm.).

## 6. Discussion

HPS is a rare respiratory syndrome in Canada, often associated with considerable morbidity and mortality, with a case fatality rate of 29% [2]. Human* Hantavirus* infection results from inhalation of aerosolized viral particles and subsequent infection of endothelial cells, resulting in capillary leakage, which in turn leads to the clinical features and laboratory anomalies seen in HPS [4–6].

Following an incubation period of up to 33 days, HPS classically begin with a 3–5-day phase of fever with nonspecific symptoms including headache, nausea, anorexia, diarrhea, abdominal pain, and malaise [1]. Typical laboratory abnormalities are sensitive but lack specificity: hemoconcentration, thrombocytopenia, granulocytosis with left shift, absence of myeloid toxic changes, and increased immunoblasts on smear [7]. A cardiopulmonary phase follows, marked by progressive respiratory symptoms and diffuse, bilateral radiographic pulmonary infiltrates. Hypoxia, hemodynamic instability, and distributive shock can progress over 24 to 48 hours and account for most case fatalities [8–10]. All three patients, following an incubation of 4-5 weeks, exhibited a similar febrile prodrome and typical laboratory abnormalities and progressed to hypoxic respiratory failure requiring cardiorespiratory support (ECMO, mechanical ventilation, or NIPPV).

The association between military activity and* Hantavirus* is not new. The first reported outbreak of “Korean hemorrhagic fever” occurred during the 1950 Korean War. Similar outbreaks were reported among US troops in Germany [11] and Korea [12], Croatian Army soldiers [13], and NATO forces in Bosnia and Herzegovina [14]. Contrary to this, autochthonous infection in the Canadian military service is uncommon. Reported cases in Canada and the United States typically involve patients from rural locations, engaged in peridomestic cleaning and agriculture or exposed in rodent-infested dwellings [2, 10, 15]. The patients in our cluster do not fit this usual profile. To our knowledge this represents the first cluster of cases among military personnel in Canada.

## 7. Conclusion

We report a rare occurrence of HPS identified in a cluster of three patients in Quebec, who shared a common epidemiological exposure. This case series outlines the importance of the consideration of HPS as a part of the differential diagnosis for severe respiratory illness. It also highlights outdoor military activity in areas of high* Hantavirus* prevalence in the rodent population, as a risk factor for HPS. Furthermore, we emphasize the importance of public health notification and its role in identifying and tracking new cases in order to mitigate further infection risk.

## Figures and Tables

**Table 1:** Results of laboratory investigations on admission.

Lab values	Patient 1	Patient 2	Patient 3
WBC (*∗*10^9^ g/L)	53.41 (4.50–11.00)	7.1 (4.8–10.8)	16.75 (4.20–10.00)
Neutrophil (abs) (*∗*10^9^ g/L)	30.72 (1.80–7.70)	2.98 (2.00–6.50)	9.23 (1.90–7.00)
Lymphocyte (abs) (*∗*10^9^ g/L)	13.58 (1.00–4.80)	1.70 (1.20–4.00)	2.86 (1.00–3.50)
Monocyte (abs) (*∗*10^9^ g/L)	7.85 (0.00–0.80)	0.43 (0.10–0.90)	2.10 (0.20–0.95)
Platelets (*∗*10^9^ g/L)	35 (140–450)	61 (150–400)	41 (150–360)
Hemoglobin (g/L)	203 (135–175)	174 (140–180)	200 (135–170)
Hematocrit (L/L)	0.640 (0.420–0.520)	0.497 (0.420–0.520)	
Creatinine (umol/L)	121 (55–110)	98 (55–105)	116 (55–105)
Band cells	26% (0–8)	24% (0–8)	7% (0–8)
Additional	Plasmacytoid lymphocytes, smudge cells, and blasts	Plasmacytoid lymphocytes	Plasmacytoid lymphocytes
